# Classification of Emotional and Immersive Outcomes in the Context of Virtual Reality Scene Interactions

**DOI:** 10.3390/diagnostics13223437

**Published:** 2023-11-13

**Authors:** Yaşar Daşdemir

**Affiliations:** Department of Computer Engineering, Erzurum Technical University, 25050 Erzurum, Turkey; yasar.dasdemir@erzurum.edu.tr

**Keywords:** electroencephalography, emotion, cybersickness, immersion, metaverse, virtual reality

## Abstract

The constantly evolving technological landscape of the Metaverse has introduced a significant concern: cybersickness (CS). There is growing academic interest in detecting and mitigating these adverse effects within virtual environments (VEs). However, the development of effective methodologies in this field has been hindered by the lack of sufficient benchmark datasets. In pursuit of this objective, we meticulously compiled a comprehensive dataset by analyzing the impact of virtual reality (VR) environments on CS, immersion levels, and EEG-based emotion estimation. Our dataset encompasses both implicit and explicit measurements. Implicit measurements focus on brain signals, while explicit measurements are based on participant questionnaires. These measurements were used to collect data on the extent of cybersickness experienced by participants in VEs. Using statistical methods, we conducted a comparative analysis of CS levels in VEs tailored for specific tasks and their immersion factors. Our findings revealed statistically significant differences between VEs, highlighting crucial factors influencing participant engagement, engrossment, and immersion. Additionally, our study achieved a remarkable classification performance of 96.25% in distinguishing brain oscillations associated with VR scenes using the multi-instance learning method and 95.63% in predicting emotions within the valence-arousal space with four labels. The dataset presented in this study holds great promise for objectively evaluating CS in VR contexts, differentiating between VEs, and providing valuable insights for future research endeavors.

## 1. Introduction

Cybersickness (CS) represents a widely recognized challenge that hinders the extensive adoption of virtual reality (VR) in real-world applications. Exploring the variables affecting CS and developing effective intervention methods to alleviate its impact is crucial. Hence, the driving force behind this study was to anticipate CS states, immersion factors, and emotional states within environments where Metaverse applications are prevalent.

The Metaverse is a digital sharing and communication platform that utilizes augmented reality (AR) and VR to immerse users in a virtual world. While this technology is not new, prior developments have significantly contributed to the emergence of novel technologies, such as VR. Researchers, particularly in fields such as virtual reality, narrative studies, and digital games, have predominantly employed the term ‘immersion’. Nevertheless, achieving a consensus on the precise definition of immersion has proven challenging due to its multidisciplinary nature and proximity to the concept of presence. In this study, more immersive virtual environments were employed to investigate symptoms of cybersickness [1,2,3].

In contrast to AR, VR fully immerses the user in a computer-generated simulation. Consequently, VR necessitates using VR devices to enhance the virtual experience. Nevertheless, ongoing developments aim to mitigate the adverse effects of this technology on users. Immersive virtual reality (IVR) involves presenting a VE so convincingly that it alters the user’s perception of the natural world, enabling complete engagement with the virtual environment [4]. The stimulus scenes in our study were chosen to be close to IVR. Despite providing a heightened sense of immersion, VR can also induce discomfort symptoms, commonly called cybersickness (CS), which has garnered significant attention in recent VR studies [5,6,7].

Datasets are widely applicable in general-purpose scenarios and are required to explain cognitive changes in the brain with virtual reality actions. Datasets find extensive utility in multifaceted, general-purpose contexts and are indispensable for elucidating cognitive alterations in response to virtual reality interactions within the neuroscientific investigation. Their primary application domains encompass Machine Learning, Artificial Intelligence, Natural Language Processing (NLP), Healthcare, Finance, and Social Sciences. In scholarly publications spanning diverse academic disciplines, it is customary to cite particular datasets as foundational evidence bolstering their research findings and practical applications. These citations exemplify the ubiquitous utilization of datasets in the realm of all-encompassing research [8,9].

This study examined the side effects of the increasingly widespread VR environments on the participants under three headings. These are cyber discomfort situations, immersion factors of VR environments, and emotion prediction made with physiological signals under VR stimuli. With these three analyses, this study differs from state-of-art studies. This study also investigated the negative impacts of virtual environments on users from the perspective of brain signals. VR has become a part of our daily life. VR technology has witnessed significant progress, benefiting the military sector with advanced training simulations and the medical field through innovative patient care and medical education applications. The main contributions of this study are (1) CS analysis in VR environments using EEG (Electroencephalography) data; (2) training the machine learning model with time and frequency features from the EEG data and presenting the classification performance of this model; (3) presenting the system immersion (engagement, engrossment, and total immersion) of virtual scenes and determining the best virtual scene capability against CS with exclusive and inclusive methods; and (4) the EEG dataset obtained with the stimuli provided by the immersive virtual environment. The major trends in simulation are:

- *Metaverse and Cybersickness Concern*: The rapidly evolving technological landscape of the Metaverse has introduced a significant concern: cybersickness (CS).
- *Academic Interest in Detection and Mitigation*: There is a growing academic interest in detecting and mitigating the adverse effects of CS within virtual environments (VEs).
- *Challenges Due to Lack of Benchmark Datasets*: The lack of sufficient benchmark datasets in this field is one of the obstacles to developing effective methodologies.

The remainder of this paper is organized as follows. Section 2 describes previous work on virtual reality, the impact of CS in VR environments, brain waves, and VR immersion. The proposed methodology is described in Section 3. Section 4 offers an analysis of CS, a factor analysis on VR immersion, a physiological ERP analysis, and an emotional estimation. Section 5 delivers the study’s conclusion.

## 2. Related Work

Sensory conflict and neural mismatches based on sensory conflict theories related to cyber disorders have been identified [10,11]. There are also theories stating that disease symptoms protect the body from toxins. For example, the theory known as “postural instability theory” hypothesizes that virtual environments can lead to feelings of sickness and discomfort [12]. These theories offer different perspectives on the causes and mechanisms of cybersickness. Identifying and understanding the underlying characteristics that lead to cybersickness can help designers develop more effective cybersickness detection models. The analyses in this study are noteworthy in this respect.

Researchers have used immersion as a technical concept for the design of virtual reality environments. Generally, they defined immersion as the “objective” and “measurable” properties of a virtual environment [13,14,15,16]. There have also been researchers who reported that immersion in mediated environments is explained by the “presence” and “flow” structures [17]. More specifically, “flow” has been defined as “the state in which individuals are so involved in an activity that nothing else seems important” [18]. On the other hand, the ”presence” structure is generally defined as “the feeling of being there” in a digital environment. It has been stated that this provides a deep sense of involvement [19]. Although presence and flow are generally accepted as optimal “states of mind”, it has been argued that immersion can be viewed as a gradual process of psychological engagement that can trigger flow and presence [20].

One of the areas of research conducted with VR is emotional state prediction studies. The emotional state in speech and video has been well studied since the mid-90s [21,22]. In human–computer interactions, emotion recognition is crucial, and Affective Computing requires data gathered in several modalities. Speech emotion databases [22], facial expression databases [23], and audiovisual databases [24,25] are available to explore the role of each method in emotion recognition. However, with the progression of technology, there is now a demand for datasets in research related to human–computer interaction. However, acquiring these datasets requires a VR environment and mobile EEG devices [26]. Both objective and subjective evaluations have been used in VR applications. Subjective evaluations for these environments were made with the help of questionnaires such as SAM, SSQ, and Virtual Reality Immersion (VRI). Objective evaluations are performed with the help of signals that the user cannot imitate (such as EEG). In this study, the objective EEG method was used. EEG studies for emotion-state predictions have been widely used [27,28]. The EEG method has received more attention for examining brain dynamics during emotional tasks [29,30].

Brain waves consist of five distinct frequency waves: delta, theta, alpha, beta, and gamma. It has been demonstrated that each brain region exhibits active alpha waves in response to changes in emotional stimuli [31]. Research has consistently shown that the classification accuracies of the alpha, beta, and gamma bands surpass those of the delta and theta bands [32].

It has been shown that virtual environment engagement is strongly associated with developing a CS using HMD in standing or sitting subjects [33]. The vestibular system perceives cybersickness as being affected by gaps between virtual and real locations [34,35,36]. While there may be statistically significant differences in the sense of presence for Content-Type, Gender, and Content-Type × gender variables, no statistically significant result may be found for any independent variables related to cybersickness [37].

Several methods are available for analyzing continuous EEG signals. The selection of the length of EEG epochs has been widely discussed [38]. In studies where the duration of VR content varies, the way the EEG is recorded and the part analyzed varies from study to study. EEG measurements can be as short as 30 s [39] or several tens of minutes [40,41], and the analysis interval differs for each researcher. For example, the authors of [42] recorded brain signals while participants watched a 2D/3D TV for 3600 s. They then compared the 60 s before and after watching the video. Dasdemir [43] measured and compared participants reading passages with and without Augmented Reality (AR) support for 120 s. Experimental results have been obtained in studies showing that individual characteristics, such as age and susceptibility, are quantitatively related to CS [8]. A CS can cause cognitive changes [44]. Therefore, studies have been conducted on the automatic detection of CS based on the patterns observed in EEG signals. Brain waves obtained using EEG have also been used to objectively detect CS [41,45,46]. Traditional machine-learning algorithms have been extensively applied to analyze EEG data for automatic stimulus scene detection [47,48,49].

## 3. Materials and Methods

**Figure 1:**
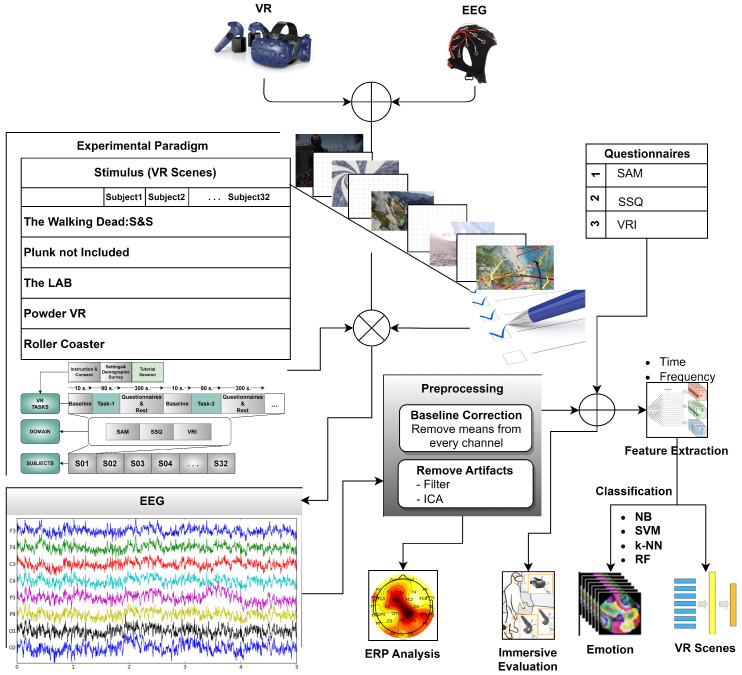
General outlines of the proposed method. First, the VR channel is used as the stimulus in this system with multi-channel input. Brain signals stimulated by the VR channel are obtained from the EEG channel. This process is carried out in accordance with the experimental paradigm. After each stimulus scene, the data in the EEG channel and the scenes in the VR channel are annotated with three different questionnaires. After the data are acquired, the data of the EEG channel are preprocessed. Finally, ERP analysis and classification of the data of the EEG channel is performed. Immersive analysis is performed for the VR channel.

An experiment was developed to compare the immersion factors of scenery, classification performances in EEG data, CS characteristics, and subjective ratings in a virtual reality environment under varied virtual task settings. EEG was used to explore the CS induction effect of the VR scenes at the neurophysiological level. In addition, subjective questionnaire ratings were collected to examine immersion and CS characteristics. Simultaneously, the subjects performed the task in a virtual environment. Figure 1 shows an overview of the VR experimental setting and flow of the experiment. This section describes the materials and methods used in the study.

EEG is often considered an inclusive measurement method in neuroscience and psychology research. EEG records electrical activity in the brain by measuring the electrical potentials produced by neurons. It provides objective data about brain activity, such as the patterns of neural oscillations, event-related potentials (ERPs), and other neurophysiological markers. Therefore, participants cannot simulate this data in experiments. Exclusive measurements with questionnaires like the Self-Assessment Manikin (SAM) [50] and SSQ are based on self-report data in which individuals rate their experiences and symptoms of CS and discomfort. It does not provide objective physiological measurements but instead captures the individual’s perception of symptoms. Objective measurements are combined with annotations of subjective questionnaire data collected from participants.

Extensive research has been conducted on the physiological changes (like EEG) and subjective assessments (Simulator Sickness Questionnaire—SSQ) associated with CS [51]. Questionnaires, such as those used in our study, are an explicit evaluation method [52,53]. The Simulator Sickness Questionnaire (SSQ), comprising 16 items on a 4-point Likert scale, is a commonly employed instrument in CS research [54]. Participants rate each item on a scale of 0 (none), 1 (slight), 2 (moderate), and 3 (severe). The SSQ is based on the following three sub-components:

(*N*) Nausea factors (e.g., general discomfort, increased salivation, sweating, nausea, difficulty concentrating, stomach awareness, burping).
(*O*) Oculomotor factors (e.g., general discomfort, fatigue, headache, eyestrain, difficulty focusing, difficulty concentrating, blurred vision).
(*D*) Disorientation factors (e.g., difficulty focusing, the fullness of the head, blurred vision, dizziness, and vertigo). Each of the three sub-components was summed. The total SSQ score was calculated as (N+O+D) × 3.74.

### 3.1. Experimental Protocol

**Figure 2:**
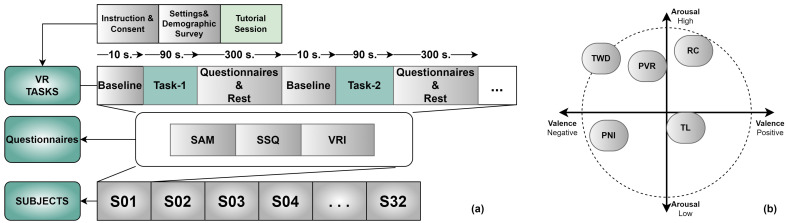
(**a**) Experimental paradigm for five different types of VR scenes (**b**) valence-arousal space.

Five virtual tasks (*N* = 32 subjects) involving immersive (audio, video, interaction) stimuli were performed in a controlled laboratory environment to evaluate immersion, the classification performance of brain oscillations, and VR sickness. Between trials, subjects completed questionnaires. If time was left between trials, they used it as a relaxation time for the subsequent trial (Figure 2). The experimental session lasted approximately 1 h. The same group of participants and devices performed all tasks.

### 3.2. Virtual Reality Environments

This study used five scenes from the VR games. These scenes were evaluated using SAM scales among 20 scenes by 15 participants who did not participate in the experiment. Because of these evaluations, a scene was chosen to fall into one of the four regions of the valence-arousal (v-a) space (Table 1). All participants were in scenes with the same brightness, contrast, and noise characteristics. These differences in scenes were used to determine the locations of the intended emotions on the v-a axis. As a result of the SAM evaluations conducted on the participants who did not participate in the experiment, their positions in the regions of the v-a space shown in Figure 2b were determined.

**Figure 3:**
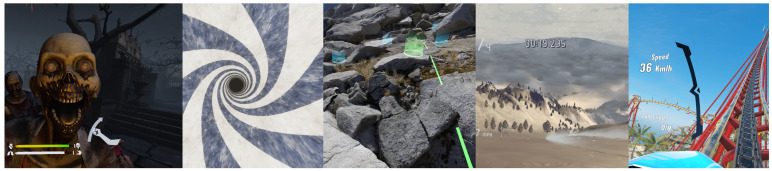
Virtual reality environments used in the experiment.

The immersion factor was high because the experiment was conducted in a six-degree-of-freedom (6DoF)–supported VR environment. Figure 3 depicts the snippets in these scenes.

**Figure 4:**
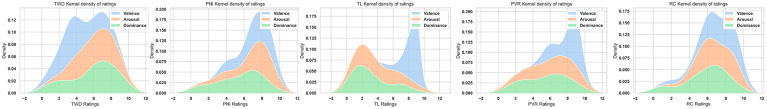
Valence, arousal, and dominance dimensions density of ratings for TWD, PNI, TL, PVR, and RC.

The central tendency of the values in the dataset appears to be above the average for valence in all stimulus scenes (Figure 4). For arousal and dominance, except for TL, the central tendency of the values also seems to be above the average value. The height of the graph indicates how dense the data points are. Since valence density is high, this indicates the presence of data points with higher values. The right and left sides of the graphs appear to be quite asymmetric. This demonstrates that data points have varying degrees of both low and high values.

### 3.3. Participants and Ethics

Before the experiment, participants were advised about safety precautions and the study procedure. Participants were informed (1) of the study purpose, (2) that they had the right to stop the experiment at any time without providing any reason, and (3) that they could stop the experiment if they felt sick or had any discomfort. All participants signed an informed consent form before undergoing the VR training. The Scientific Research and Publication Ethics Committee of Erzurum Technical University (11-2-20052021), Erzurum, TUR, reviewed and approved this study.

**Figure 5:**
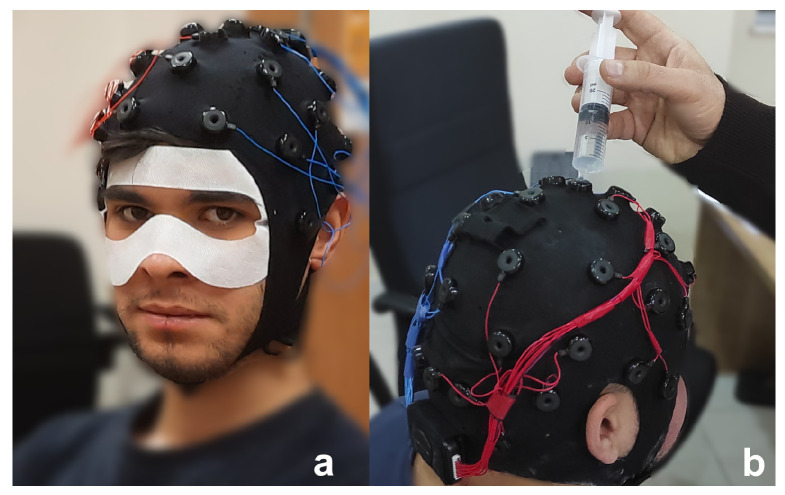
(**a**) Disposable VR Sanitary Mask compatible with HTC Vive, (**b**) preparing electrodes for contact quality.

Within the project’s scope, 35 adults volunteered to participate in this study. However, one participant used their right to withdraw from the experiment, and one participant’s data could not be used because of the experiment’s faulty hardware data collection system. The other participant was unable to complete the experiment because of extreme dizziness and nausea. Therefore, the experiment was conducted with the remaining 32 (right-handed) volunteer participants (21 males, 11 females, age range 18–30, M=21.44,SD=2.12). They had no visual or hearing impairments and normal or corrected vision. The participants were presented with a demographic survey before the experiment, which included their VR experience. Hygiene measures were taken with a Disposable VR Hygienic Mask compatible with HTC Vive. The necessary preparations were made for the contact quality of the EEG electrodes (Figure 5). On average, participants had little VR experience (M=1.53 on a 7-point Likert scale, SD=1.03, 1: none, 7: a lot); While twenty-three participants (71.87%) had never experienced VR before, nine participants (28.13%) had VR experience close to the “none” scale.

### 3.4. Experiment Apparatus

**Figure 6:**
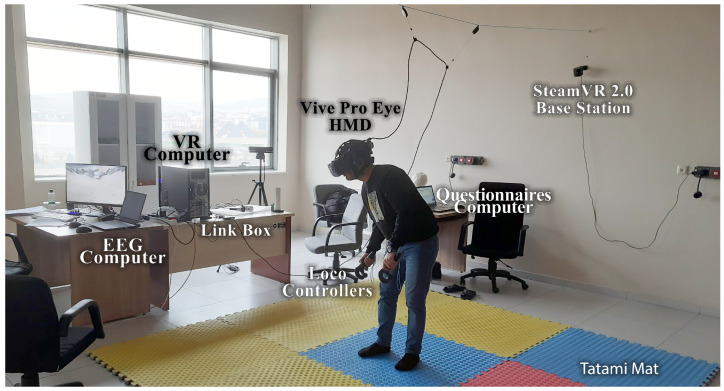
Experimental environment components.

Our experimental equipment consisted of the following parts: three computers (VR computer, EEG computer, and questionnaire computer) and an HTC (High-Tech Computer) Vive Pro Eye (HTC Corporation, Taoyuan City, Taiwan) HMD for locomotion VR scenes. The VR device was connected to a desktop computer (VR computer) with 64 GB RAM and an Intel Core i7-9700K processor running at 3.60 GHz. The Emotiv Epoc Flex device was wirelessly connected to a laptop (EEG computer) with 32 GB RAM and an Intel Core i7-11800H processor running at 2.30 GHz. The questionnaires were taken from a laptop computer (questionnaire computer) with an i5 processor and 8 GB RAM. The desktop computer had an Nvidia GeForce RTX 2080 Super GPU (8 GB), whereas the laptop had an Nvidia GeForce RTX 3080 GPU (16 GB) graphics card (Figure 6). The Emotiv Cortex API software (version 2.0) was configured (C Sharp programming) to ensure computer synchronization. The Cortex API is built on JSON and WebSockets, making accessing various programming languages and platforms easy.

**Figure 7:**
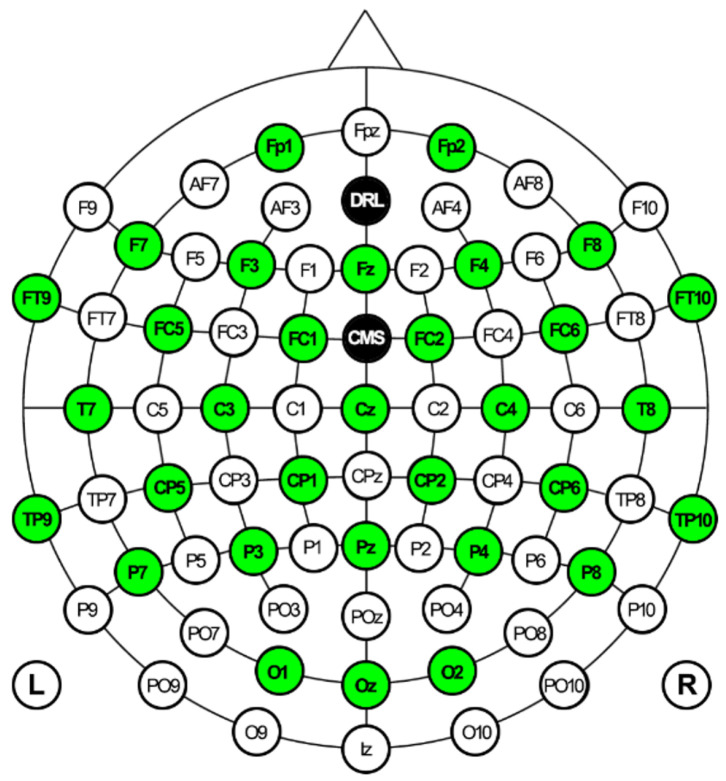
EEG electrode configuration for the current study. Green circles indicate used channels, and black circles indicate references.

EEG signals were collected by Emotiv EPOC Flex equipment, including a cap with 32 and two reference electrodes. It has been proven that the EMOTIV EPOC Flex can capture data similar to research-grade EEG systems [55]. Electrode placement follows a 10–20 system. Additionally, to prevent the HMD from exerting pressure on the anterior central electrodes, three elastic bands were used to fix the HMD, while the upper elastic band of the HMD was loose. The bands were placed between the electrodes. The EEG electrode configuration is shown in Figure 7. All electrodes were referenced to AFz (Driven-Right-Leg sensor, DRL) and FCz (Common-Mode Sensor, CMS) and grounded to the forehead. The electrodes on the cap were filled with saline solution to ensure the quality of EEG signals.

### 3.5. Feature Extraction

Feature extraction is an introductory section of classification algorithms. These features can be obtained from various domains [56]. Extracting more dominant features from an EEG signal is essential for classification. Because of their nature, EEG signals have nonlinear and spatiotemporal structures. Therefore, extracting features in the time and frequency domains from the signal [57] is significant in determining the most distinctive features for classification.

The participant’s raw EEG data were preprocessed for a better signal-to-noise ratio. Preprocessing steps were used: re-reference, resampling, filtering, artifact rejection, and epoching. This study used AFz and FCz electrodes for re-reference (Figure 7). No data are read from the references. EMOTIV products use a differential mount relative to CMS (after common mode noise cancellation). All signals reflect the potential difference between the EEG and CMS sensors. The differential mount lets you obtain relative signals between channels by subtraction: the common CMS voltage is canceled. For example, (F3 – CMS) – (F4 – CMS) = F3 – CMS – F4 + CMS = F3 – F4 is the voltage that will be observed at F3 when F4 is used as the reference level. Signals from an EEG amplifier are often subsampled for further analysis. The Emotiv EPOC Flex device used in this study was a 1024 sampling rate internally. This rate was downsampled to a 128 Hz sampling rate automatically. This value covers the typical frequency band (<50 Hz) for EEG analysis and successfully satisfies Nyquist’s signal processing theorem. The resampled EEG data were filtered to remove noise. This study applied a low-pass filter to EEG data with a cut-off frequency of 45 Hz. Baseline correction and selection of independent components were performed during preprocessing of the EEG signals. The baseline averages obtained before the experiment were extracted from all 32 channels. Electrooculogram (EOG) is one of the most important artifacts in brain signals. Independent component analysis (ICA) was applied to EEG data to eliminate this artifact and similar artifacts (e.g., blinking, muscle movements, etc.) that degrade EEG quality. ICA was applied to all channels using the MATLAB plugin EEGLab. After artifact rejection, continuous EEG data were epoched for feature extraction. This study determined the time window as 90 s (Figure 2a).

The signals are analyzed in the time domain to illustrate how the values acquired from EEG signals change over time. Changes in EEG signals sometimes exhibit a wide variance [58]. Statistical methods have also been used to reduce the effects of temporal variations in the signals. Therefore, statistical features such as the mean, variance, standard deviation, and first/second differences of the EEG data were utilized (Table 2). The first difference shows both the intensity of the signal change in the time domain and the self-similarities of the EEG signal. Another method used to analyze EEG signals in the time domain is the Hjorth parameter [59]. The Hjorth parameters (activity, mobility, and complexity) were also chosen as the feature extraction method because they have the advantage of a low computational cost (Table 2).

In the frequency domain, the relationship between the frequency and amplitude of a signal is established, and the frequency characteristics of the signal are evaluated analytically. Frequency domain information is commonly used in EEG signals with temporal and spatial dimensions. The methods of Power Spectral Density (PSD) and Spectral Entropy (SE), calculated using the Fast Fourier Transform (FFT), were employed to extract frequency domain features. PSD was calculated using Welch’s method. The signal was divided into eight segments with a 50% overlap, and each component was windowed with a Hamming window (step size of 32). So, the length of each epoch was 12 s. The PSD of each element was averaged. In our computer system, parameter learning with the Radial Basis Function (RBF) for SVM took approximately 5 min. In the case of Random Forest (RF), the learning process consumed approximately 20 min, with the number of trees set to 100 (for time domain features).

FFT can be used for various types of signal processing, such as audio, video, and EEG signals. FFT can show multiple frequency activity types in different situations (Equation (1)). Given a finite $x_{n}$ sequence of length L, Equation (1) transforms a signal of length (L≤N) into a frequency sequence dependent on the variable *k* of length *N* (with *i* = −1).

(1) $$X_{k}=\sum_{n=0}^{N-1}x_{n}e^{-i2\pi k\frac{n}{N}}k=0,1,2,\ldots ,N-1$$

where $x_{n}$ represents the time-domain signal, and $X_{k}$ is their representation in the frequency domain. Spectral Entropy (SE) provides information regarding the nonlinearity of an EEG signal. Therefore, it is used as a feature in signal processing applications [60]. The SE is the Shannon entropy of the PSD of the signal in Equation (2).

(2) $$H\left(x,f_{s}\right)=-\sum_{f=0}^{fs/2}P\left(f\right)\log_{2}\left[P\left(f\right)\right]$$

where *P* is the normalized PSD and $f_{s}$ is the sampling frequency. EEG signals were transformed into frequency components using FFT to investigate brain activity. Subsequently, Power Spectral Density (PSD) was calculated. Using the normalized PSD, the P(f) distribution function was obtained using Equation (2). Features were extracted from all 32 electrodes, yielding 320 features in the time domain and 1416 features in the frequency domain. These values represent the total computation results obtained from the EEG data acquired from 32 electrodes, as detailed in Table 2 and Equations (1) and (2). For example, in the time domain, the calculation is (32 channels × 3 Hjorth measurements) + (32 channels × 7 statistical measurements) = 320. Since normalized first/second difference values were also obtained from the statistical measurements, a total of 7 statistical measurements were acquired. In the frequency domain, it was calculated as 360 FFT measurements + 1056 Welch measurements, resulting in a total of 1416 measurements. After feature extraction, the multi-instance learning (MIL) method is applied to the feature vector [56]. Multiple-instance learning involves a set of bags, each marked as positive or negative. Each bag consists of numerous instances representing a point in the feature space. A bag is designated as positive if it contains at least one positive instance and labeled as negative if all instances within it are negative.

Measurements were obtained from participants’ survey-based assessments and ratings of their emotional states. Supervised machine learning methods were then applied to analyze the data. Naive Bayes (NB), Support Vector Machine (SVM), k-nearest Neighbors (k-NN), and Random Forest (RF) classifiers were used to classify participants’ emotional states during the virtual reality scenes. SVM is a linear or non-linear classification algorithm that finds the best-separating boundary between classes. k-NN is a simple classification algorithm that assigns data points to classes based on the majority class among their nearest neighbors. RF is an ensemble learning method that combines multiple decision trees to improve classification accuracy and robustness. NB is a probabilistic classification algorithm based on Bayes’ theorem, often used for text and categorical data classification. In addition, these classifiers were used to classify virtual scenes with brain signals. These classifiers are commonly used in EEG-based studies [28]. The parameters of all the classifiers were the same for all the operations (Table 3).

After the classification algorithms were configured, a 10-fold cross-validation (CV) approach was used to ensure performance validity. The k-fold CV approach divides a training set into k clusters. The validity of the resulting model was tested using the remaining k-1 folds as the training set. The first k-fold was used as a test set to calculate performance measures, such as accuracy. This was repeated for each k-fold, and the results were averaged. The study employed a 10-fold CV approach (k = 10).

## 4. Results and Discussion

This section presents the experimental results. Python 3.12.0, C# 7.3, MATLAB R2022b, and SPSS Version 26 were used for the programming, statistical analysis, and chart creation. First, we evaluated whether there were statistically significant differences between the virtual scenes with respect to the CS. Subsequently, factor analysis of the questionnaire items was conducted regarding the immersive effects of the scenes. Subsequently, the results of the physiological analysis are presented. This section concludes with the results of the classification of the prediction performance.

The Friedman test, SSQ, the Chi-Square test, and CS scales were used in the analyses in this section. The primary purpose of the Friedman test is to determine whether the ranking or rating differences between groups are statistically significant. SSQ helps assess the level of discomfort caused by different virtual environments. The Chi-Square test was employed to analyze whether there were significant differences in discomfort levels (CS) among different types of virtual scenes.

### 4.1. Cybersickness Status

Self-reported measures were obtained via the SSQ and VRI immersion questionnaires. This study assessed the occurrence of CS in participants within virtual environments. Table 4 presents the scores for each variable relative to CS (N,O,D, and TS). The median values of N and D are zero for the TL scene, indicating that this scene triggers less CS. It also stands out in terms of the CS level for RC and *D*.

The non-parametric Friedman test was used to evaluate the SSQ. The Friedman test results with the chi-square test showed significant differences in score severity for the virtual scene varieties based on their SSQ Scales. The results of the Friedman test indicate that virtual scenes affect the CS levels. In terms of CS (SSQ): *N* ($\chi^{2}\left(4\right)=30.46,p<0.01$); *O* ($\chi^{2}\left(4\right)=31.16,p<0.01$); *D* ($\chi^{2}\left(4\right)=44.84,p<0.01$); and TS ($\chi^{2}\left(4\right)=37.61,p<0.01$). In the case of the SSQ, significant differences were found in the variables.

The Friedman test result was significant (the scales differed significantly depending on the CS type). It is an omnibus test statistic. Hence, it is ambiguous which VR scenes have the most significant effects on CS. Pairwise comparisons were performed using the Conover post-hoc test to determine which scenes were substantially different from TS. Multiple pairwise comparisons showed statistically significant differences between the TL and other scenarios regarding the TS level (Table 5). The RC and TWD also showed statistically significant differences (p<0.05).

**Figure 8:**
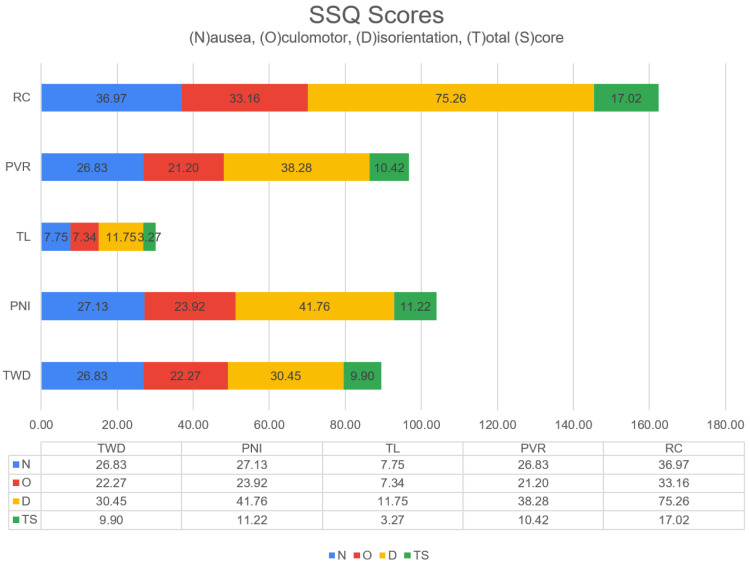
Total simulator sickness scores.

Among the virtual scenes, The LAB caused less nausea than the other scenes (Figure 8). RC and PNI scenes caused more cyber discomfort in the vestibular system.

### 4.2. Immersion Analysis

The ARI questionnaire [61] was modified for VR (Virtual Reality Immersion, VRI) for immersion analysis (Table 6). Cheng et al. [62] proposed an immersion model that organizes immersion into three levels: “engagement”, “engrossment” and “total immersion”. This model was validated using Exploratory Factor Analysis (EFA) and Confirmatory Factor Analysis (CFA). The Cronbach α values for each level and substructure ranged from 0.70 to 0.92, which were entirely satisfactory. Additionally, statistical analysis validated the immersion model’s proposed hierarchical structure’s dependability and good model fit [62].

In cases where individual scores were found by summing the answers given to the questions on a scale containing k questions, Cronbach’s alpha coefficient was used to find the similarity and closeness of the questions (Equation (3)). Because the scale’s reliability is calculated as 0.6≤α<0.8, the scale is quite reliable [63].

(3) $$\alpha =\frac{k}{k-1}\left[\frac{\sigma_{t}^{2}-\sum \sigma_{i}^{2}}{\sigma_{t}^{2}}\right]$$

*k* is the number of questions, $\sigma_{t}^{2}$; the variance of the total column; $\sigma_{i}^{2}$; and the variance of each variable. In the “Engagement” level analyses, KMO=0.805 was calculated because of the test performed to understand whether the sample size was suitable for factor analysis. When this value is above 0.50, it is considered sufficient [64]; between 0.8–0.9, it is classified in the “excellent” category. Additionally, because of the Bartlett test, ($\chi^{2}\left(105\right)=1313.95,p<0.01$) was found, and this finding showed that the correlations between the items were sufficiently significant for EFA. Because of the EFA, it was determined that the “engagement” level, consisting of 12 items, consisted of a 5-factor structure, and these five factors explained 70.83% of the total variance. Table 7 lists the results for other levels.

The factor items obtained for engagement were as follows:

“**A1**: The VR application we employed captured my attention”
“**A15**: I felt confident since I knew how to use the VR application”
“**A11**: The time I spent on the activity was more than I expected”
“**A16**: I felt that I could use the VR application to find the information I wanted”

The factor items obtained for the engrossment were as follows:

“**B8**: I often forgot the passage of time during the activity”
“**B5**: I often felt that I was really in charge of the activity”

The factor items obtained for total immersion were as follows:

“**C4**: The activity felt so authentic that it made me think that the virtual characters/objects existed for real”
“**C3**: During the activity, I felt that I was the protagonist”
“**C12**: All of my senses were totally concentrated on the activity”

### 4.3. Physiological and Emotional Analyses

While ERPs were not the focus of this study, the term was used metaphorically to describe the observed electrical brain responses during our EEG measurements. Research results show how ERP topographies recorded during the presentation of emotional stimuli are modulated by emotional tasks. Emotional tasks are instrumental in investigating participant responses to distinct emotional stimuli (VR scenes). The utilization of ERP topographic maps facilitates a more nuanced examination of neural activity during the execution of these tasks. Such an approach allows for a comprehensive comprehension of the precise cortical regions implicated in response to specific emotional stimuli and the temporal dynamics of these implications.

**Figure 9:**
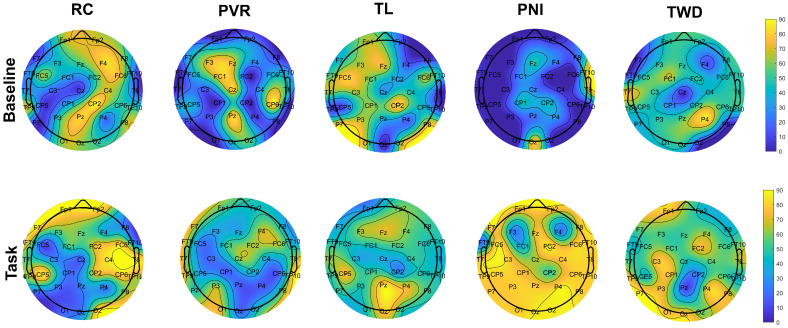
The temporal distribution of the topographic maps revealed by the spatiotemporal segmentation analysis (filtered ERP peak times).

Figure 9 shows a comparison of the ERP topographies of the baseline and tasks. It shows a topographic graphic of peak times during tasks, which are the baseline of the ERP. This was implemented for a low-pass-filtered ERP system. A windowed “sinc” function and a 45 Hz low-pass filter were used. The waveform focusing on the time series is primarily shaped at low frequencies. Low-pass filtering eliminates high-frequency activity at an event-related potential. Averaging over trials is already a low-pass filter because high-frequency activity is less likely to be preserved than low-frequency activity when averaging over multiple trials. These are not topographical maps that show voltage or any other measure of activity. Instead, they are topographic maps that show peak time latency. An ERP was created for each channel, and the maximum voltage values in the “0–90” s range were used for the emotional task. This process was repeated for the baseline. Therefore, the most significant positive or negative deviation was not sought. What we care about in this study we focused on latency, the time at which the maximum occurred.

The RC was the most nauseating task. According to the baseline of this stimulus scene, activity was observed in the frontal, right parietal, right occipital, and left central parietal regions of the brain. In the RC task, no activity was observed in the visual cortex, parietal, or left-front central regions. In the visual cortex, the peak time latency was more prominent in the occipital region in the PNI, TL, and TWD.

Studies examining the discomfort experienced by participants due to HMDs are conducted at the level of EEG bands [65]. In particular, the correlation with CS was positive in some of the EEG bands. The delta, theta, alpha, and beta band powers were calculated, and the correlation with CS was positive [40,66]. ERP analysis of 44 participants in all bands of the EEG showed a positive correlation with CS, especially in the frontal and central lobes [67]. It has also been shown that cyber disease severity is highly correlated in some locations (especially frontoparietal and temporal) in EEG frequency bands reflecting CS-related brain activity [8].

**Figure 10:**
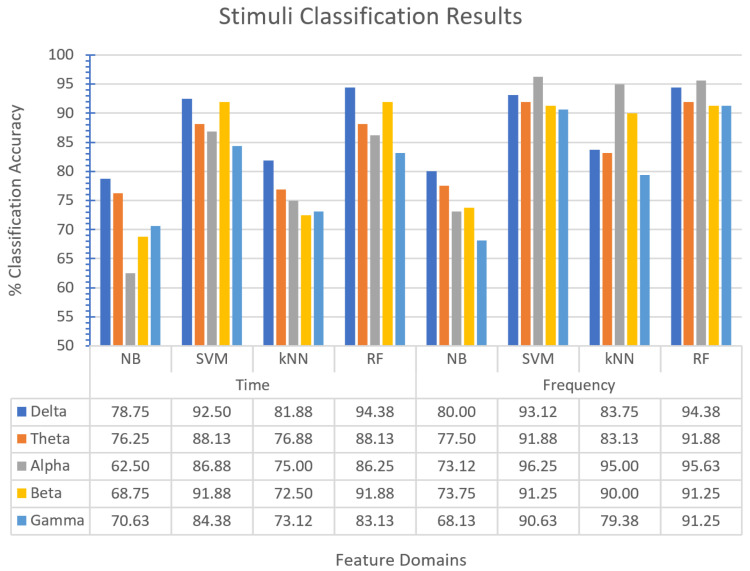
Virtual scene classification results.

**Figure 11:**
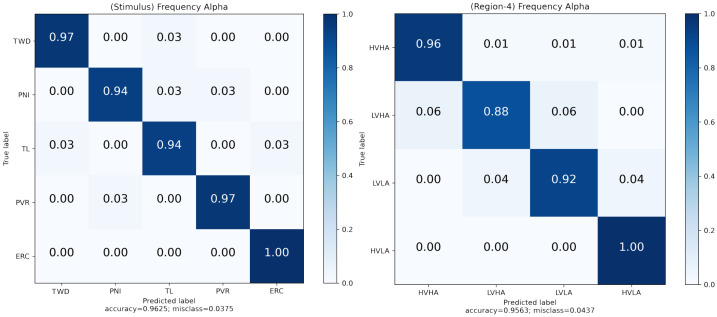
Highest accuracy results (**left**: stimulus, **right**: regions).

Multi-classification was performed with virtual scenes used as stimuli. High performance was achieved using EEG-assisted classification. In terms of the classification algorithms, the SVM and RF algorithms performed well (Figure 10). In this way, the effect of multi-instance learning comes to the fore. When the classification performances were examined in terms of bands, the highest accuracies were obtained in the alpha (SVM, 96.25%) and delta (RF, 94.38%) bands. The alpha band was dominant in the EEG bands (Table 8). Regarding the feature domains, the frequency domain’s classification performance is generally high (Figure 11).

Table 9 and Table 10 list the metric data for the highest performance. The SVM algorithm showed the highest performance for stimulus classification in the frequency domain and alpha band. Notably, the RC scene exhibited a 100% performance in classifier discrimination (Table 9). Similarly, the highest accuracies for the four-region classification were found in the frequency domain and alpha band using the RF algorithm (Table 10).

Table 11 shows the classifier methods used in the state-of-the-art, the highest accuracy of the classifier, and the corresponding method. While cybersickness levels are classified in other studies, in our proposed study, the achievements of emotion classification (according to four regions in the valence-arousal space) are presented along with the classification of stimuli. The promising success of the proposed method according to the number of classes can be seen in Table 11.

The results were examined under three headings: Cybersickness situations, entrainment factors of VR environments, and emotion prediction with physiological signals under VR stimuli. Cyber disturbance situations were examined with statistical analysis, and RC and TWD stimuli showed statistically significant differences. Additionally, TL caused the most minor nausea, while RC caused the most cyber discomfort at all levels. In the immersion analysis, the immersion factors of VR environments were investigated. Again, in this analysis, the proposed method was confirmed with EFA and CFA analyses. As a result of these analyses, the most critical factors for three immersion levels (engagement, engrossment, and total immersion) are presented. Factors A1, A11, A15, and A16 stood out for the engagement level. Factors B5 and B8 stood out for Engrossment, and C3, C4, and C12 for Total Immersion level. The topographic map that emerged during the baseline and task moments was first obtained in physiological and emotional prediction analyses. The peak time latency regions stimulated by the stimuli in the participants are presented in Figure 8. In addition, the classification performance of five-label stimulation and four-label emotion regions was at the highest levels in the state-of-the-art. While the True Positive rate for RC was 100%, the overall performance was 96.25%.

All results were validated with 10-fold cross-validation (Table 9 and Table 10). To test the validity of the dataset, leave-one-out cross-validation (LOOCV) was also used (Table 12). For Stimulus only, LOOCV was performed without splitting the dataset into bands.

## 5. Conclusions

This study observed that individuals vulnerable to motion sickness experienced more CS in immersive virtual environments, which caused motion sickness. Self-reported measures (SSQ and VRI) and EEG-based physiological measures were also used. From the feature extraction, it is clear that frequency features are more distinctive in the classification results than time features. Regarding cybersickness, the RC scene was more effective as a stimulus on all N, O, D, and TS scales. The TL scene had less impact on all CS scales and caused less cybersickness because it consisted of authentic natural images, and the task in this scene did not require much engagement. When ERP analysis was examined, more peak moments were observed in the right hemisphere for the RC stimulus. For the TL, more peak moments were observed in the occipital region. However, peak moments were observed in almost all cortical areas in the PNI. Approximately 96% success was achieved in the classification performance using EEG recordings.

Based on the findings of this study, aggregating CS results by age group, sex, VR content, and device type may provide useful information for objectively measuring CS. Additionally, the dataset obtained in this study can be used as a benchmark for different machine learning or signal processing methods. A more precise understanding of the properties of brainwaves and CS through these studies will provide safe guidance for VR users. Nevertheless, they will also aid in developing hardware and content that mitigates this inconvenience. 

## Figures and Tables

**Table 1:** VR software that includes virtual scenes.

Short Name	Excerpt’s Source	Task
RC	Epic Roller Coaster	Accomplishing a roller coaster circuit
TWD	The Walking Dead: Saints & Sinners	Immersing oneself in an environment inhabited by zombies
PVR	Powder VR	Efficiently completing a timed ski course, racing against the clock
TL	The LAB	Observing Vesper Peak, a prominent mountain located in Snohomish County, Washington, USA
PNI	Plunk not included	Encountering optical illusions within the surroundings.

**Table 2:** Time-domain features used in the study.

#	Measures	Category	Formula
1	Power	Statistical	$P=\frac{1}{N}\sum_{-\infty }^{\infty }{\left|x(t)\right|}^{2}$
2	Mean	Statistical	$\mu =\frac{1}{N}\sum_{t=1}^{N}x\left(t\right)$
3	Standard deviation	Statistical	$\sigma =\sqrt{\frac{1}{N}\sum_{t=1}^{N}{\left(x\left(t\right)-\mu \right)}^{2}}$
4	Normalized first difference	Statistical	$d_{1n}=\frac{1}{N-1}\sum_{t=1}^{N-1}\left|x(t+1)-x(t)\right|\sigma_{x}$
5	Normalized second difference	Statistical	$d_{2n}=\frac{1}{N-2}\sum_{t=1}^{N-2}\left|x(t+2)-x(t)\right|\sigma_{x}$
6	Activity	Hjorth	$A=\frac{\sum_{i=1}^{N}{\left(x\left(t\right)-\mu \right)}^{2}}{N}$
7	Mobility	Hjorth	$M=\sqrt{\frac{var(\frac{dx(t)}{dt})}{var(x(t))}}$
8	Complexity	Hjorth	$C=\frac{M(\frac{dx(t)}{dt})}{M(x(t))}$

**Table 3:** Classifier algorithm parameters.

Classifiers	Params	Values	Notes
NB	batchSize	100	This value strikes a balance between computational efficiency and model stability.
	Use Kernel Estimator	False	We set the use of a Kernel Estimator to False because our data may not have the characteristics that benefit from kernel density estimation.
SVM	c	1.0	The choice of C = 1.0 for the Support Vector Machine’s regularization parameter is a common starting point.
	epsilon	1.0 × 10^−12^	We selected an epsilon value of 1.0 × 10^−12^ for numerical stability and precision in the SVM model.
	kernel	RBFKernel-C 250007-G 0.01	These values were chosen through experimentation and tuning to optimize model performance.
k-NN	K	3	A small k value may help the model capture local patterns in the data.
	searchAlgorithm	LinearNNSearch	The linear nearest neighbor search was chosen as the search algorithm for k-NN.
RF	Iterations	100	This number provides a good trade-off between model complexity and performance based on our prior experience.
	Depth	0 ${}^{a}$	This value allows the tree to capture intricate patterns in the data.
	Features	0 ${}^{b}$	Set to 0 so that all existing features are taken into account when splitting the tree.
	Seed	1	We used a seed value of 1 for reproducibility.
	Bag size percent	100	All samples in the dataset are considered for bagging in methods like RF.

${}^{a}$ for unlimited, ${}^{b}$ int(log2(#predictors) + 1 was used for 0).

**Table 4:** Results obtained for the SSQ questionnaire throughout the study.

#	Scenes ${}^{a}$	N ${}^{b}$			O			D			TS		
		Mean	Median	Std.Dev.	Mean	Median	Std.Dev.	Mean	Median	Std.Dev.	Mean	Median	Std.Dev.
1	TWD	26.83	19.08	28.86	22.27	7.58	23.74	30.45	0.00	47.90	29.69	11.22	34.32
2	PNI	27.13	28.62	22.26	23.92	30.32	19.61	41.76	34.80	38.28	33.66	37.40	26.86
3	TL	7.75	0.00	12.26	7.34	7.58	8.58	11.75	0.00	20.91	9.82	3.74	12.18
4	PVR	26.83	28.62	19.29	21.56	15.16	19.52	38.28	13.92	42.62	31.44	24.31	26.19
5	RC	36.97	28.62	31.26	33.16	34.11	26.45	75.26	55.68	35.37	51.07	44.88	40.58

${}^{a}$ TWD: The Walking Dead: Saints Sinners; PNI: Plunk not included; TL: The LAB; PVR: Powder VR; RC: Roller Coaster. ${}^{b}$ (N) ausea; (O) culomotor; (D) isorientation; (T) otal (S) core.

**Table 5:** Multiple pairwise comparisons for VR Scenes.

	TWD	PNI	TL	PVR	RC
TWD	1.000	0.123	0.025	0.286	0.003
PNI	0.124	1.000	2 × 10^−4^	0.556	0.165
TL	0.025	2 × 10^−4^	1.000	0.001	6 × 10^−7^
PVR	0.286	0.556	0.001	1.000	0.062
RC	0.003	0.165	6 × 10^−7^	0.062	1.000

**Table 6:** VRI (Virtual Reality Immersion) questionnaire.

Immersion Level	Factors	Items #
Engagement	Interest	6
	Time investment	6
	Usability	6
Engrossment	Emotional Engagement	5
	Focus of attention	7
Total Immersion	Presence	8
	Flow	4

**Table 7:** EFA results for each level.

Immersion Level	KMO	EFA	Total Variance (%)	α
Engagement	0.805	$\chi^{2}$(105) = 1313.95, *p* < 0.01	69.33	0.784
Engrossment	0.901	$\chi^{2}$(66) = 1305.76, *p* < 0.01	65.65	0.880
Total Immersion	0.822	$\chi^{2}$(66) = 1178.78, *p* < 0.01	72.79	0.861

**Table 8:** Alpha band performance error metrics.

	MAE ${}^{a}$	RMSE	RRSE (%)
Frequency-Alpha (Stimulus)	0.243	0.3209	80.189
Frequency-Alpha (Regions)	0.255	0.3219	80.268

${}^{a}$ MAE: Mean Absolute Error, RMSE: Root Mean Squared Error, RRSE: Root Relative Squared Error.

**Table 9:** Feature frequency alpha classification results (for stimulus).

TP Rate	FP Rate	Precision	Recall	F-Measure	ROC Area	Class
0.969	0.008	0.969	0.969	0.969	0.970	TWD
0.938	0.008	0.968	0.938	0.952	0.987	PNI
0.938	0.016	0.938	0.938	0.938	0.973	TL
0.969	0.008	0.969	0.969	0.969	0.972	PVR
1.000	0.008	0.970	1.000	0.985	0.996	RC

**Table 10:** Feature frequency alpha classification results (for regions).

TP Rate	FP Rate	Precision	Recall	F-Measure	ROC Area	Class
0.965	0.013	0.988	0.965	0.976	0.975	HVHA
0.875	0.014	0.875	0.875	0.875	0.951	LVHA
0.920	0.015	0.920	0.920	0.920	0.985	LVLA
1.000	0.016	0.944	1.000	0.971	0.988	HVLA

**Table 11:** Comparison of performance with the state-of-the-art.

References	Methods ${}^{a}$	Number of Class	Highest Accuracy
Liao et al. [44]	MLP, CNN, **LSTM**	5 (SSQ levels)	82.83%
Hadadi et al. [68]	RBF-SVM	2 (sick, non-sick)	71.00%
Yang et al. [69]	RF	2 (sick, non-sick)	84.00%
Kundu et al. [70]	LSTM	4 (CS levels:none, low, medium, high)	94.00%
Qu et al. [71]	LSTM	3 (CS levels:none, slight, severe)	96.85%
Munoz et al. [72]	DT	3 (low, medium, high)	70.00%
The proposed study	NB, K-NN, RF	5 (Stimulus)	96.25%
	**RBF-SVM**	4 (SAM-Regions)	95.63%

${}^{a}$ The classifier in bold showed the best accuracy, CNN: convolutional neural network, DT: decision tree, K-NN: k-nearest neighbor, LSTM: long short-term memory, MLP: multilayer perception, NB: naive Bayes, RBF-SVM: radial basis function SVM, RF: random forest, SVM: support vector machine.

**Table 12:** Average LOOCV results for valence without splitting the signal into bands (for stimulus).

Stim.	MAE ${}^{a}$	RMSE	RAE (%)	ACC
TWD	0.084	0.257	9.045	0.956
PNI	0.038	0.168	8.938	0.952
TL	0.016	0.283	12.385	0.947
PVR	0.078	0.304	13.690	0.954
RC	0.031	0.263	10.564	0.985

${}^{a}$ MAE: Mean Absolute Error, RMSE: Root Mean Squared Error, RAE: Relative Absolute Error, ACC: Accuracy.

## Data Availability

The VREMO data will be published at https://eegdatasets.erzurum.edu.tr, and the dataset requests will be considered by the corresponding author within the scope of the license agreement. The dataset will be available on 10 June 2024.
